# Assessing Real-World Racial Differences Among Patients With Metastatic Triple-Negative Breast Cancer in US Community Practices

**DOI:** 10.3389/fpubh.2022.859113

**Published:** 2022-05-24

**Authors:** Ruoding Tan, Lourenia Cassoli, Ying Yan, Vincent Shen, Bann-mo Day, Edith P. Mitchell

**Affiliations:** ^1^Genentech Inc., San Francisco, CA, United States; ^2^Gastroesophageal Center, Thomas Jefferson University Hospital, Philadelphia, PA, United States

**Keywords:** triple-negative breast cancer, racial differences, African American, real-world, community practices, Flatiron Health

## Abstract

**Objective:**

Real-world data characterizing differences between African American (AA) and White women with metastatic triple-negative breast cancer (mTNBC) are limited. Using 9 years of data collected from community practices throughout the United States, we assessed racial differences in the proportion of patients with mTNBC, and their characteristics, treatment, and overall survival (OS).

**Methods:**

This retrospective study analyzed de-identified data from 2,116 patients with mTNBC in the Flatiron Health database (January 2011 to March 2020). Characteristics and treatment patterns between AA and White patients with mTNBC were compared using descriptive statistics. OS was examined using Kaplan-Meier analysis and a multivariate Cox proportional hazards regression model.

**Results:**

Among patients with metastatic breast cancer, more AA patients (23%) had mTNBC than White patients (12%). This difference was particularly pronounced in patients who lived in the Northeast, were aged 45–65, had commercial insurance, and had initial diagnosis at stage II. AA patients were younger and more likely to have Medicaid. Clinical characteristics and first-line treatments were similar between AA and White patients. Unadjusted median OS (months) was shorter in AA (10.3; 95% confidence interval [CI]: 9.1, 11.7) vs. White patients (11.9; 95% CI: 10.9, 12.8) but not significantly different. After adjusting for potential confounders, the hazard ratio for OS was 1.09 (95% CI: 0.95, 1.25) for AA vs. White patients.

**Conclusions:**

The proportion of patients with mTNBC was higher in AA than White mBC patients treated in community practices. Race did not show an association with OS. Both AA and White patients with mTNBC received similar treatments. OS was similarly poor in both groups, particularly in patients who had not received any documented anti-cancer treatment. Effective treatment remains a substantial unmet need for all patients with mTNBC.

## Introduction

African American (AA) women with breast cancer have long experienced significant health disparities. Despite having similar breast cancer incidence as White women, AA women are more likely to be diagnosed with late-stage breast cancer and have an approximately 40% higher mortality rate than White women (1).

Breast cancer is heterogeneous in nature, with prognosis and survival varying considerably by subtype. Compared with other breast cancer subtypes, triple-negative breast cancer (TNBC) is a particularly aggressive form of breast cancer. It is more likely to arise in younger women, be of higher histologic grade, present at a more advanced stage, relapse earlier, and show worse prognosis (2–5). Previous epidemiological studies have found the incidence of TNBC to be twice as high among AA women as White women (2, 4, 6). The disproportionally higher incidence of the TNBC subtype in AA women may contribute to the racial disparity in breast cancer mortality.

While there is abundant evidence of racial differences in the prevalence of TNBC, it remains unclear whether there are racial differences in treatment patterns and clinical outcomes between AA and White patients. To date, real-world studies comparing racial differences in treatments and clinical outcomes between AA and White TNBC patients have yielded mixed findings. While some studies reported shorter survival for AA women with TNBC compared with White women (2, 7–11), other studies found no evidence of a survival difference (12–17). Research in this area has often been limited to single-institution data with small sample sizes, regional data from a single state, or databases with limited clinical and treatment variables.

Factors contributing to racial differences in TNBC prevalence and potential differences in outcomes include biological, social, economic, and environmental factors. Emerging preclinical and clinical data suggest that TNBC in AA women may have a uniquely aggressive biology. Some studies comparing the genetic risk factors in TNBC by race found AA patients have a higher rate of pathogenic variants (18) or different gene expression patterns (19). However, the extent to which genetic risk factors contribute to the observed racial difference in incidence and outcomes is unclear. Other biological features that may contribute to the difference in incidence and prognosis between AA and White women with TNBC include differences in the tumor immune microenvironment; expression of breast cancer-associated cancer stem cells; prevalence of obesity, which is known to be linked to an increased risk of metabolic disorder; and tissue inflammation (20).

Beyond biological factors, many sociodemographic factors are associated with health outcomes in breast cancer, including TNBC. Among them, status and type of health insurance coverage are important factors commonly studied and have far-reaching implications for care including time to diagnosis and quality of treatment. Lack of insurance and type of insurance (i.e., Medicaid vs. private insurance) were found to be significantly associated with worse survival in patients with TNBC (21). Other social determinants of health, such as geographic location (rural vs. urban, disadvantaged vs. average neighborhood) and family structure (married vs. unmarried), may also predict the quality of care that patients receive, which ultimately influences outcomes (22, 23).

In this study, we analyzed recent data with wide geographic representation of community oncology practices across the United States. We assessed racial differences across the care continuum of patients, ranging from diagnosis to treatment to survival. Our goal was to better understand how patient and disease characteristics, treatments received, and differential access to care may underlie racial differences in prevalence and outcomes of mTNBC in community oncology practices.

## Materials and Methods

### Data Source: Flatiron Health Database

The Flatiron Health Database is an oncology-focused, real-world database primarily generated from OncoEMR^®^, a proprietary electronic health record (EHR) system used by community oncologists throughout the USA. This retrospective, observational study evaluated data from Flatiron Health's longitudinal, demographically, and geographically diverse database derived from electronic health record data from more than 280 community-based cancer treatment clinics and academic centers, representing more than 2.2 million active US patients with cancer. The database is composed of patient-level structured and unstructured data, curated via technology-enabled abstraction. Structured data (e.g., patient demographics, drugs ordered) are prespecified by the software and captured during routine patient care. Data were de-identified and provisions were made to prevent re-identification for patient confidentiality. The Flatiron database contains detailed documentation of treatments, biomarkers, and clinical outcomes.

### Study Cohort

**Figure 1:**
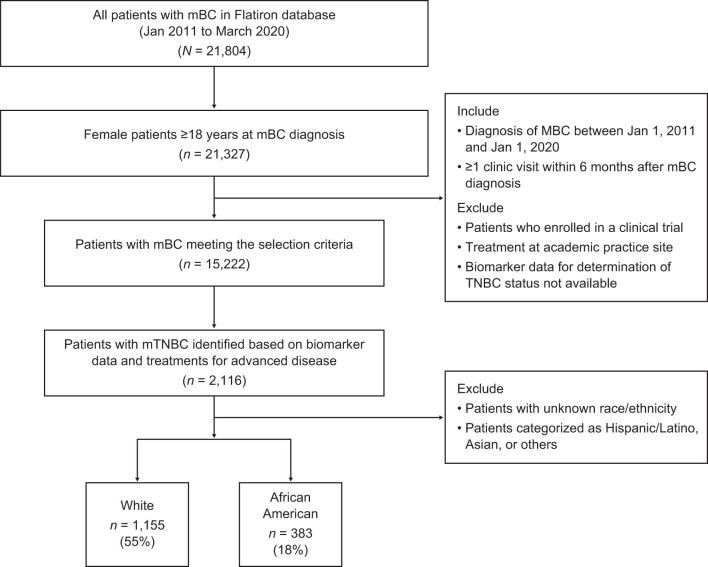
Patient identification in the Flatiron database. mBC, metastatic breast cancer; mTNBC, metastatic triple-negative breast cancer; TNBC, triple-negative breast cancer.

The study cohort (Figure 1) was comprised of all AA and White women (≥18 years of age) with a confirmed diagnosis of metastatic breast cancer (mBC) between January 1, 2011 and January 1, 2020. Patients were required to have data collected during at least one visit within 6 months of their metastatic diagnosis and identifiable information to assess tumor estrogen receptor (ER), progesterone receptor (PR), and human epidermal growth factor receptor 2 (HER2) expression. To ensure that only real-world patients treated in a community oncology setting were included in the analysis, patients who received an investigational drug in any line of therapy or were treated in an academic setting were also excluded. In line with previous studies (24), the cohort of patients with mBC was subsequently categorized by breast cancer subtype (HER2+, ER+/PR+, TNBC). The final cohort of patients with mTNBC was identified for outcomes analysis if patients had ≥ 1 negative result for ER and PR and ≥ 1 negative or equivocal result for HER2 status. Among them, those who received HER2-targeted and/or hormonal therapy for mBC were further excluded from the analysis. Patients of races other than AA or White were also excluded from further analysis.

### Study Variables and Outcomes

Race/ethnicity was collected through routine oncology clinical care. Other patient sociodemographic characteristics included age, geographic location (state and region), and insurance status at the time of mBC diagnosis. Disease characteristics included stage at initial diagnosis, metastatic disease type, number and location of metastases, and Eastern Cooperative Oncology Group (ECOG) performance status (the most recent within 1 year of mBC diagnosis).

The primary clinical outcome for this study was overall survival (OS), which was measured from the time of metastatic diagnosis date until time of death. Patients not reported as having died at the time of the analyses were censored at the last activity date or the study end date.

Time to treatment initiation (TTI) was also evaluated as duration between date of metastatic diagnosis and first-line treatment start date. First-line treatments for patients with mTNBC were captured and grouped into broad categories (i.e., single-agent chemotherapy, combination chemotherapy, targeted therapy and/or cancer immunotherapy, and other treatments) and drug class (i.e., taxanes, platinums, anthracyclines, cyclophosphamide, antimetabolites, and microtubule inhibitors).

### Statistical Analysis

Descriptive statistics were generated for all study variables, including means, standard deviations, medians, and ranges for continuous variables, and frequencies and counts for categorical variables. Comparisons of patient characteristics and treatment patterns between AA and White patients were conducted using *t*-tests. For categorical variables, Chi-square tests were used when ≤ 20% of the groups for comparison had expected frequencies <10; otherwise, the Fisher's exact test was used.

The proportion of each mBC subtype was described as the proportions of TNBC, HER2+, and ER+/PR+ phenotypes out of all mBC patients in the Flatiron Enhanced Data Mart. To further assess racial differences in the proportion of patients with mTNBC in the study cohort, a ratio was constructed by dividing the proportion of mBC that was mTNBC in AA patients by the proportion of mBC that was mTNBC in White patients. These ratios were calculated for the overall patient cohort as well as for patients in relevant subgroups determined by state of residence, age group, geographic region, insurance status, and disease stage at initial diagnosis.

The Kaplan-Meier method was used to estimate median OS in patients with mTNBC, with log-rank tests performed to compare the unadjusted difference in OS between AA and White patients. A Cox proportional hazards model was used to adjust for the potential effect of key prognostic variables on OS including age (<65 years, ≥65 years), region (Northeast, South, West, Midwest missing), type of occurrence (recurrent, de novo, unknown), ECOG performance status at mTNBC diagnosis (0–1, ≥2, unknown), and treatment (received or not). Differences in OS between AA and White patients were also evaluated within subgroups of patients stratified by age group, insurance type, location, ECOG performance status, disease stage at diagnosis, sites or number of metastases, receipt of treatment, and type of first-line treatment.

## Results

Of the 21,804 patients diagnosed with mBC, 1,538 eligible patients with mTNBC were identified (Figure 1).

### Proportion of mTNBC Subtype

**Figure 2:**
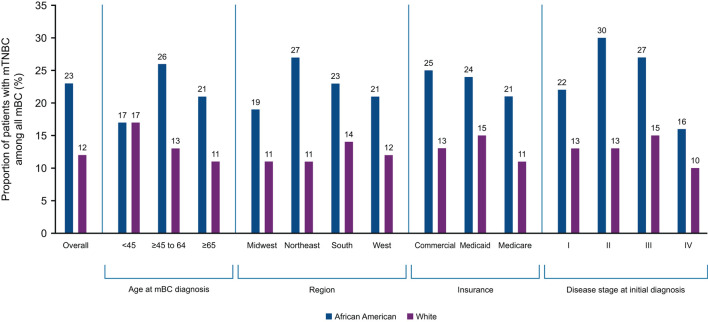
Proportion of mBC that is mTNBC in African American and White patients by key characteristics. mBC, metastatic breast cancer; mTNBC, metastatic triple-negative breast cancer.

**Figure 3:**
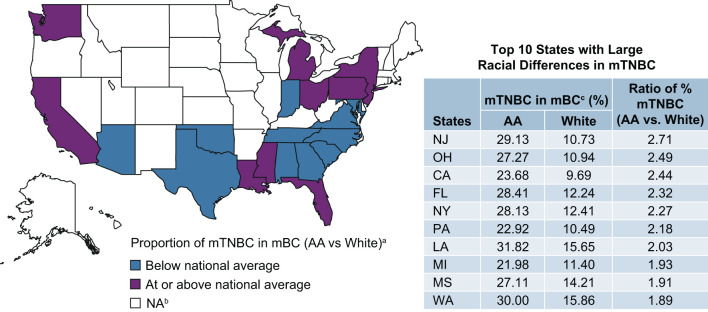
Proportion of mBC that is mTNBC in AA and White patients by US state. ^a^Proportion of mTNBC in mBC is 1.86 (22.88%/12.29%) times higher in AA than in White patients (national average using Flatiron data). ^b^Data from 18 states were excluded due to small N (<10) of reported mTNBC cases. ^c^Source: Flatiron Health Data. AA, African American; mBC, metastatic breast cancer; mTNBC, metastatic triple-negative breast; NA, not applicable.

Twenty-one US states reported mTNBC cases by race. Among all mBC patients in the Flatiron data, the proportion of mTNBC subtype in AA patients was approximately 1.9 times that of White patients (AA vs. White: 23 vs. 12%). Racial differences in the proportion of mTNBC (AA vs. White) were particularly pronounced among patients aged 45–65 years (26 vs. 13%; Supplementary Table 1), treated in the Northeast (27 vs. 11%), with commercial insurance (25 vs. 13%), and with initial diagnosis at disease stage II (30 vs. 13%). The magnitude of difference in the proportion of TNBC in AA compared with White patients was similar regardless of insurance status (Figure 2). Seven states (New Jersey, Ohio, California, Florida, New York, Pennsylvania, and Louisiana) had more than twice as many AA than White mBC patients with the mTNBC subtype. Louisiana had the highest proportion of mTNBC in AA (32%) mBC patients, compared to all states with available data (Figure 3).

### Patient Characteristics

Patient baseline demographics, clinical characteristics, and first-line treatments are shown in Table 1. Compared with White patients at the time of mBC diagnosis, AA patients were younger (mean: 60 years vs. 63 years; *p* <0.001), more likely to have Medicaid coverage (10 vs. 3%, *p* < 0.001), and less likely to have Medicare coverage (18 vs. 26%, *p* = 0.003). Geographic location differed significantly between AA and White patients, with more AA women residing in the South (68 vs. 44%, *p* < 0.001). Clinical and cancer characteristics were similar between AA and White patients, including the distribution of disease stage at initial diagnosis, disease recurrence, ECOG performance status, and the sites and number of metastases.

**Table 1:** Characteristics of patients with mTNBC by race.

	**White (*n* = 1,155)**	**African American (*n* = 383)**	***p*** **-value**
**Demographic Characteristics**			
**Mean age at metastatic diagnosis, years (SD)**	63 (12)	60 (12)	<0.001
**Insurance** ^a^			
Commercial	469 (41)	146 (38)	0.423
Medicaid	39 (3)	38 (10)	<0.001
Medicare	301 (26)	70 (18)	0.003
Missing	337 (29)	138 (36)	0.014
**Region**			
Northeast	239 (21)	62 (16)	<0.001
Midwest	200 (17)	33 (9)	
South	505 (44)	261 (68)	
West	176 (15)	18 (5)	
Missing	35 (3)	9 (2)	
**Clinical Characteristics**			
**Disease stage at initial diagnosis**			
0–II	474 (41)	141 (37)	0.172
III–IV	585 (51)	215 (56)	
Unknown	96 (8)	27 (7)	
**Disease type**			
De novo	309 (27)	98 (26)	0.641
Recurrent	751 (65)	258 (67)	
Unknown	95 (8)	27 (7)	
**ECOG PS at metastatic diagnosis**			
0 or 1	562 (49)	170 (44)	0.280
≥2	118 (10)	38 (10)	
Unknown	475 (41)	175 (46)	
**Number of metastases^b^, median (range)**	2 (1–6)	2 (1–6)	0.960
**Sites of metastasis** ^b^			
CNS/Brain	364 (32)	108 (28)	0.248
Bone	580 (50)	195 (51)	0.859
Liver	410 (35)	127 (33)	0.441
Lung	579 (50)	205 (54)	0.274
Lymph node	544 (47)	179 (47)	0.949
Other	401 (35)	127 (33)	0.621
**Treatment Characteristics**			
**Patients with documented treatment**	869 (75)	287 (75)	
**First-line regimens—treatment grouping**			
Single-agent chemotherapy^c^	477 (55)	151 (53)	0.780
Chemotherapy combination treatment^d^	318 (37)	114 (40)	
Targeted therapy or cancer immunotherapy	69 (8)	20 (7)	
Other therapy	<10	<10	
**Time to first-line treatment for mTNBC**			
Median time to first-line treatment from metastatic diagnosis, months (range)	1 (<1–40)	1 (<1–36)	0.939
Time to treatment <1 month	469 (54)	154 (54)	
Time to treatment 1 to <2 months	216 (25)	71 (25)	
Time to treatment 2 to <3 months	72 (8)	27 (9)	
Time to treatment ≥3 months	112 (13)	35 (12)	

*Data are n (%) unless otherwise stated*.

*CNS, central nervous system; ECOG PS, Eastern Cooperative Oncology Group performance status; mTNBC, metastatic triple-negative breast cancer; SD, standard deviation*.

a *Insurance type was collected at the time of metastatic diagnosis. Patients may have had multiple insurance types*.

b *Sites and number of metastases were measured at the time of metastatic diagnosis. Patients may have had metastases at multiple sites*.

c *Antimetabolites were the most frequently used single-agent chemotherapy (African American: 24%; White: 23%), and capecitabine was the most used agent within this class*.

d *Platinum-based treatments were the most frequently used chemotherapy combination treatments (African American: 20%; White: 23%)*.

### Time to Treatment Initiation and First-Line Treatment

TTI and first-line treatment were also similar in AA and White patients. Overall, 25% of patients in each racial group had no documentation in the database of receiving anticancer treatment after mBC diagnosis (Table 1).

Among patients who received first-line treatment, more than half initiated treatment <30 days after diagnosis, and median TTI did not differ by race. More than half of the patients received single-agent chemotherapy as their first-line treatment (AA: 53%; White: 55%), with capecitabine being the most frequently administered chemotherapy agent within this class. Combination chemotherapy was used to treat 37% of patients with similar frequencies between AA and White patients; platinum-based therapy was the most frequently administered combination therapy (Table 1).

### Overall Survival

**Figure 4:**
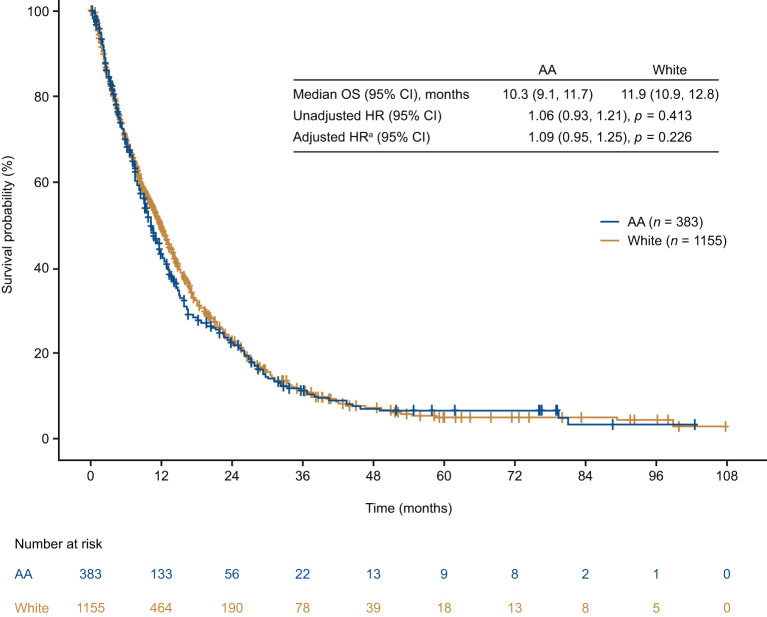
Overall survival from time of mTNBC diagnosis in African American and White patients. ^a^Model adjusted for age, geographic region, insurance type, de novo vs. recurrent mBC, ECOG performance status, and receipt of treatment. AA, African American; CI, confidence interval; HR, hazard ratio; OS, overall survival.

**Figure 5:**
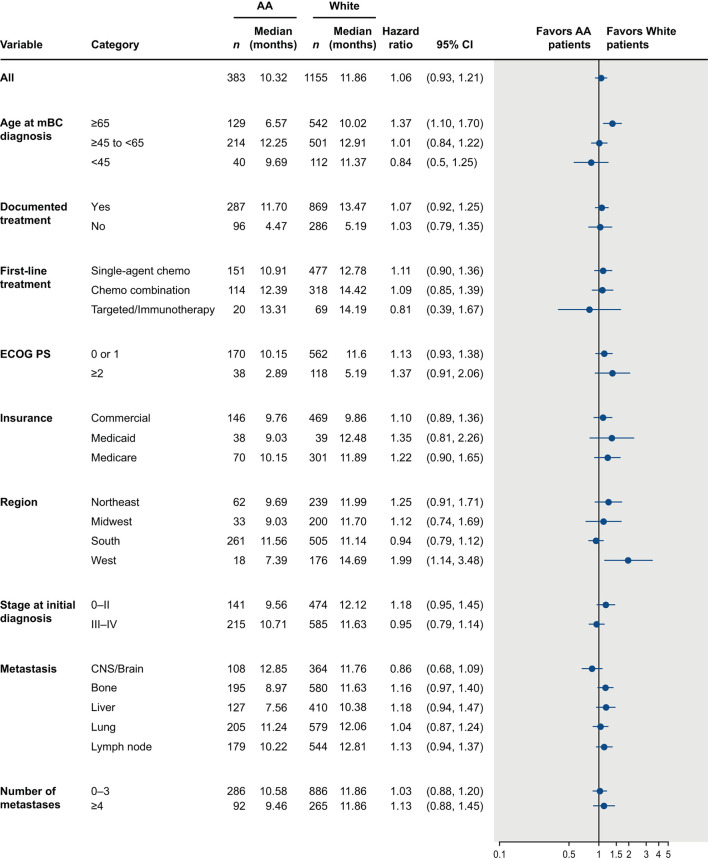
Overall survival from time of mTNBC diagnosis in African American and White patients by patient subgroups. AA, African American; CI, confidence interval; CNS, central nervous system; ECOG PS, Eastern Cooperative Oncology Group performance status; HR, hazard ratio; mBC, metastatic breast cancer; mTNBC, metastatic triple-negative breast cancer.

The median OS was <1 year in both AA and White patients (AA: 10.3 months, 95% confidence interval [CI]: 9.1, 11.7; White: 11.9 months, 95% CI: 10.9, 12.8). Although median OS was numerically shorter in AA patients, this difference was not statistically significant (unadjusted hazard ratio = 1.06 [95% CI: 0.93, 1.21; *p* = 0.413]; Figure 4). After adjusting for key prognostic factors, AA patients did not appear to have a significantly greater risk of death compared with White patients (adjusted hazard ratio = 1.09 [95% CI: 0.95–1.25; *p* = 0.226]). We also assessed differences in OS between AA and White patients in subgroups defined by age, insurance type, region, ECOG performance status, disease stage at diagnosis, sites and number of metastases, receipt of treatment, and type of first-line treatment (Figure 5). The results consistently showed no association between race and OS, regardless of subgroups, except for patients located in the West.

## Discussion

Racial differences in mTNBC prevalence, disease characteristics, and clinical outcomes have been well-documented (19, 20). Most of these data, however, have been derived from single-center studies or from population-based surveillance system data. Single-center data can lack generalizability and often have small sample sizes. While population-based surveillance system databases provide large sample sizes and reliable epidemiologic data, these databases tend to have limited information on patient clinical characteristics and treatment patterns. In this observational study, we sought to gain greater insight into racial differences in the proportion of mBC that is mTNBC and outcomes in real-world settings; we did this by interrogating the Flatiron Health database, a large database which includes data from demographically and geographically diverse community oncology practices in the United States.

We first examined racial differences in the percentage of mBC cases with the triple-negative phenotype in a cohort of mBC patients from January 2011 to March 2020. We found AA women with mBC were more likely to be diagnosed with the mTNBC subtype than White women. The difference between AA and White patients was more pronounced in mBC patients who were younger, had commercial insurance, were initially diagnosed at an earlier stage, and lived in several geographic “hotspot” areas such as Louisiana. These results are not only consistent with data derived from various cancer registries (4, 6, 25), but also expand on these prior findings by comparing the proportion of the mTNBC subtype among all mBC cases by AA and White race in subgroups defined by region, age, insurance type, and disease stage.

We further assessed whether race was associated with clinical outcomes within the cohort of patients with mTNBC, a question that remains inadequately addressed due to the conflicting real-world evidence generated so far. Data generated from this mTNBC patient cohort suggest that OS was poor among the entire cohort and did not differ significantly between AA and White patients overall and within subgroups stratified by demographic, clinical, and treatment characteristics. In contrast to previous reports of population-based surveillance that found significantly increased mortality from TNBC in AA women compared with White women (2, 7), our findings are consistent with more recent data from the National Comprehensive Cancer Network Outcomes database (12), the Carolina Breast Cancer Study database (14), and several single institutions across the United States (13, 15–17). A recent analysis of data from the Surveillance, Epidemiology, and End Results (SEER) program demonstrated greater mortality from non-metastatic TNBC in AA women compared to White women, but the disparity varied by patient, disease, and treatment characteristics (11). The inconsistency among studies is likely a result of the multiple factors that contribute to outcomes including biological factors, as well as various social, economic, and environmental factors that are known to affect access to care. These factors must be carefully considered to accurately discern differences in outcomes. Further it must also be noted that variation is likely to occur in population-based vs. community-based vs. clinical trial-based data.

Our data show that patients who received care in the community oncology setting faced a high unmet need regardless of race. This is not only reflected by the poor OS, but also by the lack of evidence of first-line treatment initiation in a notable proportion of patients in both racial groups. Consistent with a previous study that examined treatment patterns in real-world patients with mTNBC treated in the community setting (10), we found one in four patients with mTNBC in both racial groups had missing documentation of anticancer treatment in the Flatiron database. It is important to note that some patients with mTNBC may have forgone treatment due to poor performance status and concerns about treatment tolerance. However, since reasons for lack of treatment were not documented, this cannot be verified. It is also possible that some patients received cancer treatments outside of the Flatiron network that were not captured in the database.

The present analysis has several limitations that are inherent to electronic health record-based retrospective observational studies. First, there was limited information on the social determinants of health in this dataset, which reduced our ability to further assess how socioeconomic disparities interplay with racial disparity in patients with mTNBC. The adjustment for insurance coverage and geographic location in the regression model can be considered as a proxy for socioeconomic status; however, more granular, multilevel data to characterize access to care, quality of care, and socioeconomic well-being are needed to better understand factors and mechanisms driving racial disparity in mTNBC. Secondly, incomplete/missing information on performance status and comorbidity burden may also lead to misclassification and unmeasured confounding. Thirdly, while this study has improved generalizability compared to existing racial disparity research in mTNBC, which is predominantly conducted in regional or single institution settings (2, 7, 13–16), our study cohort was restricted to patients receiving care in the community oncology setting, and findings may not be representative of those treated in other settings (e.g., academic, research institutions) or those who do not engage with the healthcare system. For patients treated in the community oncology setting, there may be disparities in the usage of the Flatiron proprietary data system across urban, suburban, and rural communities. The selection of the Flatiron network for clinical record keeping may also have been preferred by certain types of community practices, leading to potential selection bias that could affect patient outcome. Lastly, our conclusions must be interpreted with respect to how race/ethnic information is captured. In contrast to registries such as SEER or National Program of Cancer Registries, race/ethnicity information in the Flatiron database is self-reported and voluntary. This data acquisition approach may not reliably account for multi-ethnic individuals or other genetic ancestry heterogeneity. In this study cohort, approximately 11% of patients had missing race/ethnicity information, a proportion that is larger than in other registry databases. Overall, we believe these limitations are outweighed by the study's strengths including its large, geographically diverse, well-characterized cohort assessing racial differences in patient outcomes across the care continuum from diagnosis to treatment to OS outcomes.

In summary, we found large differences between AA and White women in the proportion of the mTNBC subtype among women with mBC, especially in younger patients and patients who lived in geographic “hotspots”. Time to treatment initiation and type of first-line treatment were similar between AA and white patients. OS was poor among the entire cohort and did not differ significantly between racial groups, suggesting that mTNBC is an aggressive disease, regardless of race. Effective treatment remains a substantial unmet need for patients with mTNBC. Considering the lack of racial differences in this patient cohort, prospective studies are needed to further elucidate the biological differences that may have predictive or prognostic significance for AA patients with mTNBC.

## Data Availability Statement

The datasets presented in this article are not readily available because the data that support the findings of this study have been originated by Flatiron Health, Inc. These de-identified data may be made available upon request, and are subject to a license agreement with Flatiron Health; interested researchers should contact DataAccess@flatiron.com to determine licensing terms. Requests to access the datasets should be directed to DataAccess@flatiron.com.

## Author Contributions

RT, LC, and EM contributed to the conception and design. YY, VS, and B-mD performed data analysis and interpretation. All authors wrote the manuscript and approved the final version. All authors are accountable for all aspects of the work.

## Funding

The authors declare that this study received funding from Genentech, Inc. The funder had the following involvement: study design, data analysis, interpretation of data, and writing of this article.

## Conflict of Interest

RT, LC, YY, VS, and B-mD are employed by Genentech, Inc. and hold stock in F. Hoffmann-La Roche, Ltd. The remaining author declares that aside from the medical editing support she received, the research was conducted in the absence of any commercial or financial relationships that could be construed as a potential conflict of interest.

## Publisher's Note

All claims expressed in this article are solely those of the authors and do not necessarily represent those of their affiliated organizations, or those of the publisher, the editors and the reviewers. Any product that may be evaluated in this article, or claim that may be made by its manufacturer, is not guaranteed or endorsed by the publisher.
