# Supplementary material for: Assessing Real-World Racial Differences Among Patients With Metastatic Triple-Negative Breast Cancer in US Community Practices
Source: Front Public Health. 2022 May 24;10:859113. doi: 10.3389/fpubh.2022.859113 (PMC9171051; doi:10.3389/fpubh.2022.859113)
Supplement: Supplementary file 1 [file Table_1.docx]

***Supplementary Material***

1. **Supplementary Data**

**Supplementary Table 1**. Frequencies (%) of TNBC Subtype According to Race and Age

|  | **White** | | **African American** | |
| --- | --- | --- | --- | --- |
|  | **Patients with mBC, *n*** | **Patients with TNBC, *n* (% of patients with mBC)** | **Patients with mBC, *n*** | **Patients with TNBC, *n* (% of patients with mBC)** |
| All patients | 9,400 | 1,155 (12.3) | 1,674 | 383 (22.9) |
| Age at metastatic diagnosis |  |  |  |  |
| <45 years | 674 | 112 (16.6) | 231 | 40 (17.3) |
| ≥45 to <65 years | 3,771 | 501 (13.3) | 832 | 214 (25.7) |
| ≥65 years | 4,955 | 542 (10.9) | 611 | 129 (21.1) |

*mBC, metastatic breast cancer; mTNBC, metastatic triple-negative breast cancer.*
